# Early physical therapy intervention in gynaecological surgery: “Case series”

**DOI:** 10.1016/j.ijscr.2018.09.051

**Published:** 2018-10-10

**Authors:** Ébe dos Santos Monteiro Carbone, Mayara Ronzini Takaki, Maria Gabriela Baumgarten Kuster Uyeda, Marair Gracio Ferreira Sartori

**Affiliations:** a Urogynecology and, Department of Gynecology, Escola Paulista de Medicina (EPM), Universidade Federal de São Paulo (UNIFESP), São Paulo, SP, Brazil; b Department of Gynecology, EPM-UNIFESP, São Paulo, SP, Brazil

**Keywords:** Physical therapy, Early ambulation, Gynecology

## Abstract

**Purpose:**

To implement a physical therapy intervention protocol targeting patients admitted for gynaecological surgery to the gynaecological ward of XXXX Hospital.

**Method:**

A prospective, cross-sectional and observational study was conducted with women admitted to the gynaecology ward, from June 2014 through June 2015. The study was divided into three phases with data on admissions to the gynaecology ward. A total of 565 women were included, corresponding to phases I (197), II (178) and III (190). The physical therapy staff implemented an early ambulation protocol as well as a mobility assessment.

**Results:**

The physical therapy protocol was implemented, and the rate of adherence was 100%. All participants received preoperative instruction on the importance of early mobilisation. On postoperative day 1, the participants in phase I walked a mean of 77.4 m. Following implementation of the physical therapy protocol, the walked distance increased to 292.6 m in phase II, followed by a slight decrease to a mean of 233 m in phase III.

**Conclusions:**

The physical therapy protocol could be implemented, and the patients’ adherence was satisfactory. Early ambulation can be optimised, and the participants began ambulation starting at 13 h after surgery.

## Highlights

• The distance walked increased following the implementation of the protocol.
• Early ambulation led to longer walked distances.
• Longer walked distances led to shorter hospital stays.
• The longer the surgery elapsed time, the shorter the distance walked.
• The pain resulted in shorter walked distances and indicated that hospital stay might be longer.

## 1 Introduction

In the 1990s, the Danish surgeon Henrik Kehlet developed the notion of fast-track surgery. This was the basis for the Enhanced Recovery After Surgery (ERAS) protocols, which represent an evidence-based multimodal treatment to improve postoperative recovery and reduce perioperative stress, morbidity and mortality rates as well as length of hospital stay [[1], [2], [3], [4]].

Early mobilisation is an integral part of systematic efforts to improve recovery protocols. The need for physical therapists to indicate early mobilisation within the first 24 h after surgery is a subject of much discussion [[5], [6], [7], [8], [9], [10]].

Based on the aforementioned considerations, the aim of the present study was to implement an early physical therapy intervention protocol at the gynaecology ward of XX (XX), Federal University of XX (XX) and investigate the impacts of starting ambulation as early as possible.

## 2 Material and methods

The present study was based on the European multicentre project ERAS and on the Aceleração da Recuperação Total Pós-Operatória/Acceleration of Total Postoperative Recovery (ACERTO) Project [11].

The result was the Otimizando a Recuperação durante a Internação na Ginecologia Atendimento à Mulher de modo Integral/Optimising Recovery during Admission to Gynaecology–Integral Women’s Health Care (ORIGAMI) Project, which was designed to facilitate communication between the Department of Gynaecology and XXXXXXX units and chairs.

The present study was subjected to and approved by the research ethics committees of XX and XX under unified ruling no. 1,763,134. And the research was registered with ResearchRegistry.com, with Research Registry Unique Identifying: Numberesearchregistry4276. The research work has been reported in line with the PROCESS criteria [12].

The present prospective, cross-sectional and observational study was conducted with women admitted from June 2014 through June 2015 to the gynaecology ward of HSP, namely, the university hospital. A total of 697 women were admitted for surgery during this period, and 565 were included in the physical therapy study.

**Fig. 1:**
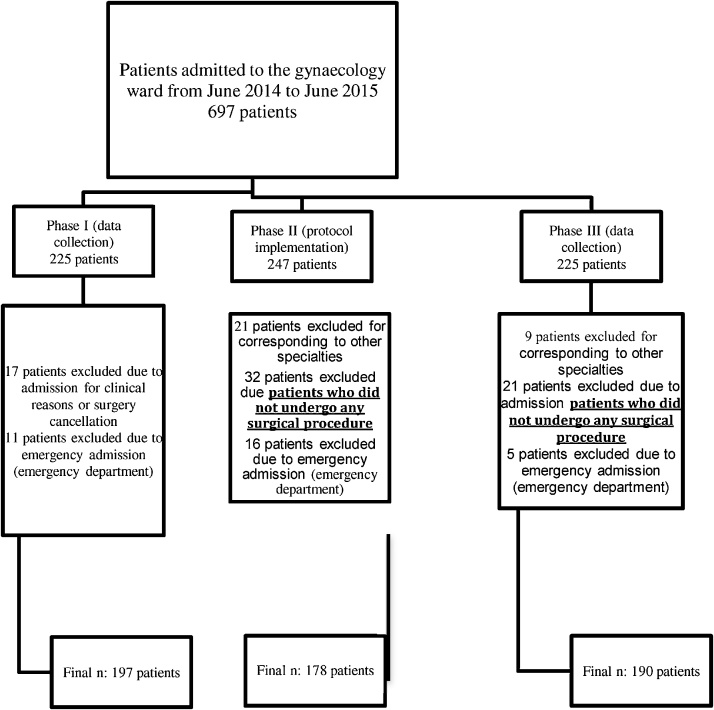
Flowchart representing the participants of the ORIGAMI Project–physical therapy intervention.

The study was divided into three phases. The total sample, 697 participants, was allocated to phases I (n = 225), II (n = 247) and III (225) (Fig. 1).

After exclusions, 197 participants from phase I, 178 from phase II and 190 from phase III remained for analysis. Therefore, the final sample comprised 565 women. The reasons for exclusion were:

Absence of surgical procedure due to: lack of material, lack of clinical conditions to undergo surgery, diseases in which there was no surgical indication, absence of gynecological disease and first care in the emergency room, in other words, emergency or emergency hospitalizations.

Before data collection, a multidisciplinary team including gynaecologists, anaesthesiologists, nurses, physical therapists and nutritionists was convened to define the variables of interest and methods for data collection in compliance with the recommendations made by the ACERTO Project.^11^ The study was divided into three phases.

### 2.1 Phase I

Collection of admission data before any intervention. In this phase, we gathered data entered in the data collection forms while the medical, nursing, physical therapy and psychology staff members had no knowledge about the project. In this phase, we performed a prospective, cross-sectional and observational evaluation of variables related to early postoperative ambulation relative to women admitted to the gynaecology, from July through September 2014. A total of 225 patients were admitted to the ward during this period.

### 2.2 Phase II

This phase encompassed October and November 2014 and January 2015. The main problems detected in phase I were analysed. Interdisciplinary meetings with surgeons, anaesthesiologists, nurses, physical therapists and nutritionists were held to define new care delivery routines for inpatients.

The project was presented to the Department of Gynaecology of XXXXX and the various involved chairs, units and residents.

The physical therapy staff launched the early ambulation and mobility assessment protocol. An explanatory brochure was prepared and then delivered to and discussed with patients upon admission.

A total of 247 patients were admitted, and 178 were included in the physical therapy study.

Three physical therapists were available at the gynaecology ward to implement the protocol.

### 2.3 Phase III

This phase was carried out during April-June 2015, i.e., after the ORIGAMI Project was launched. All suggested changes were made. We collected the same data as in phase I to investigate possible changes following the implementation of the protocol (phase II). During this phase, 225 patients were admitted to the ward, and 190 were included in the physical therapy study.

The physical therapy staff introduced the following routines in the gynaecology ward:

• Preoperative instruction, involving delivery of a manual with information on the importance of early mobilisation
• Onset of ambulation 6 h after surgery

The data collection forms were affixed to the sides of the beds, and each physical therapist responsible for care delivery entered the required information. During phase I, the physical therapists were unaware of the study aims and were instructed to provide the standard postoperative care according to the patients’ individual needs. As a rule, postoperative care included assessment and respiratory and motor physical therapy without any pre-set plan or uniform approach among the physical therapists. The analyses were based on the time to onset of ambulation.

#### 2.3.1 Preoperative physical therapy intervention

• Before surgery, explain the details of the procedure to patient, encouraging her to walk and eat as soon as possible
• Perform functional assessment, with normal functioning defined as an absence of difficulty in performing certain gestures and activities of daily living [13].
• Instruct the patient on physical therapy procedures to be performed during the immediate postoperative period
• Investigate possible risk of postoperative respiratory and vascular complications
• Instruct the patient to move her feet as soon as possible, even when still on the gurney

#### 2.3.2 Postoperative physical therapy intervention

• Check the routine laboratory tests. For cases lacking all tests, assessment was based on the patient’s clinical condition and medical evaluation
• Platelets >150,000/mm,
• Haemoglobin>10 g%
• Calcium <6 mg/dL
• Potassium >3 mEq/L
• Sodium >130 mEq/L [14]
• Check the vital signs
• BP (blood pressure): 80 mmHg > systolic arterial pressure (SAP) <150 mmHg, 60 mmHg > diastolic arterial pressure (DAP) < 90 mmHg)
• SpO_2_ (oxygen saturation) >90%
• Heart rate <130 bpm (beats per minute)
• Temperature 36° > T < 36.9°
• Respiratory rate <20 bpm (breaths per minute) [15]
• Check the patient’s condition: absence of dizziness, nausea, vomiting, headache
• Raise the bed to 60 ° for at least 30 min, and look for signs of postural hypotension [16].
• With the patient sitting, perform metabolic exercises for 60 seconds
• Have the patient sit on the side of the bed for at least 3 min, and look for signs of postural hypotension, such as dizziness, nausea, blurred vision, fatigue, headache, cognitive impairment, or neck or shoulder pain; in such cases, the patients were instructed to lie down to revert the symptoms, and the corresponding information was entered in the data collection form
• Ultra-early mobilisation: help the patient sit on the very same day surgery was performed and with a shorter rest period

State that there are no conflicts of interest and no sources of funding

Phase I was designed to identify the deficiencies in physiotherapeutic care. After this phase, a systematized service protocol was established, based on phase I information, and was implemented in phase II. In phase III, the protocol was already in place, after all the training obtained during phase II. Thus, in phase III the physiotherapeutic approach was uniform among the professionals

### 2.4 Sample characteristics

Table 1 lists the participants’ characteristics. The average age was 51, 50 and 49 years in phases I, II and II, respectively, without significant differences among phases.

**Table 1:** Description of the participants’ characteristics—age, race and comorbidities.

	Phase I	Phase II	Phase III	p¹
age; mean ± SD	51.07 ± 14.35 (n = 197)	50.62 ± 15.43 (n = 178)	49.6 ± 14.65 (n = 190)	0.608²
**Race (N)**	192	177	185	
Asian	1 (0.52%)	2 (1.13%)	2 (1.08%)	0.287
White	118 (61.46%)	120 (67.8%)	119 (64.32%)	
Native American	3 (1.56%)	0	0	
Black	8 (4.17%)	10 (5.65%)	13 (7.03%)	
Mixed	62 (32.29%)	45 (25.42%)	51 (27.57%)	
**Comorbidities (N)**	197	178	190	
Heart disease	8 (4.06%)	5 (2.81%)	5 (2.63%)	0.684
DLP	22 (11.17%)	19 (10.67%)	28 (14.74%)	0.423
DM	28 (14.21%)	39 (21.91%)	34 (17.89%)	0.152
SAH	87 (44.16%)	73 (41.01%)	73 (38.42%)	0.517
SAH and DM	28 (14.21%)	32 (17.98%)	29 (15.26%)	0.592
Hyperthyroidism	2 (1.02%)	0	1 (0.53%)	0.402
Hypothyroidism	26 (13.2%)	19 (10.67%)	19 (10%)	0.578
HIV	2 (1.02%)	2 (1.12%)	2 (1.05%)	0.995
CKF	1 (0.51%)	1 (0.56%)	1 (0.53%)	0.997
Rheumatologic	3 (1.52%)	9 (5.06%)	2 (1.05%)	0.027
Asthma	0	0	4 (2.11%)	0.019
Kidney disease	0	0	2 (1.05%)	0.138
Neoplasm	1 (0.51%)	11 (6.18%)	3 (1.58%)	0.002
Stroke	1 (0.51%)	3 (1.69%)	1 (0.53%)	0.387
Chagas	0	0	1 (0.53%)	0.372
Sickle cell	0	0	1 (0.53%)	0.372
Hepatitis B	1 (0.51%)	0	0	0.392
Hepatitis C	2 (1.02%)	1 (0.56%)	2 (1.05%)	0.856
Parkinson	0	1 (0.56%)	0	0.337
DVT	4 (2.03%)	3 (1.69%)	2 (1.05%)	0.739
Smoking	23 (11.68%)	25 (14.12%)	24 (12.63%)	0.776

(SAH) systemic arterial hypertension; (DM) diabetes mellitus; (DLP) dyslipidaemia; (HIV+) human immunodeficiency virus; (CKF) chronic kidney failure; (DVT) deep vein thrombosis. (1) Chi-square test. (2) ANOVA F test.

Approximately one third of the participants in all three phases had cancer. Only 2% of the participants in phases I and II and 0.5% in phase III were admitted to the intensive care unit (ICU) after surgery. The incidence of complications was low in all study phases. With a sample of 565 patients, the groups were homogeneous and also regarding the surgical size and the access route. Thus, there was no statistical difference regarding the curirgic procedure, when the three phases were analyzed

### 2.5 Statistical analysis

The mean values of continuous variables were compared between pairs of phases with Student’s *t*-test (ANOVA for three or more groups) or the non-parametric Mann-Whitney test (Kruskal-Wallis test for three or more groups) when the normality assumption was not met. Categorical variables were compared by means of the chi-square test [17].

Correlations between the times analysed are presented as dispersion plots with the corresponding Pearson’s correlation coefficient value [18] Calculations were performed with statistical software R 3.3.3 [1,19].

## 3 Results

A total of 565 patients were included in the present study, including 197 in phase I, 178 in phase II and 190 in phase III.

The rate of adherence to the physical therapy protocol was 100%. All participants received preoperative instruction on the importance of early mobilisation and performed lower limb movements as soon as the effects of anaesthesia subsided.

We analysed the time to onset of ambulation (hours), duration of initial ambulation (minutes), and walked distance (meters) during the immediate postoperative period (IPO) and on postoperative day 1 (PO1) relative to the other surgical variables.

### 3.1 Time to onset of ambulation

The times to onset of ambulation after surgery decreased from one phase to the next: 16.65 h in phase I, 13.85 h in phase II and 13.50 h in phase III. The time to onset of ambulation was approximately 13 h in phases II and III, and 25% of the participants in phase II began ambulation approximately 6 h after the end of surgery. It is worth noting that the full physical therapy staff was available in phase II and that the physiotherapeutic conduct was intensified; physicians and nutritionists also collaborated during this phase.

The length of hospital stay decreased from 77.79 h in phase I to 72.36 h in phase II. The time to onset of ambulation also significantly decreased over this period (from 16.65 to 13.85 h) (Table 2).

**Table 2:** Comparative analysis of time to onset of ambulation and length of hospital stay among study phases.

Variables	Phase I	Phase II	Phase III	p
	(n = 197)	(n = 178)	(n = 190)	
Time to ambulation Mean/SD	16.65 ± 8.87	13.85 ± 8.02	13.50 ± 7.62	<0.001¹
Median/IQR	16.67 [13.5–20.75]	15.21 [6.71–18.43]	13.83 [7–18.17]	<0.001¹
Length of hospital stay Mean/SD	77.79 ± 65.13	72.36 ± 48.78	60.26 ± 31.68	0.011¹
Median/IQR	52.85 [50.13–76.8]	52.22 [49.23–74.83]	51.65 [48.53–71.3]	0.011¹

SD: standard deviation; IQR: interquartile range; (1) Kruskal-Wallis test; (2) Fisher’s exact test.

The mean time to onset of ambulation in phase I was 16 h. Based on these data, we sought to establish whether the length of hospital stay was related to ambulation starting before or after 16 h since surgery, as shown in Table 3.

**Table 3:** Length of hospital stay of participants who began ambulation before or after 16 h since surgery.

Phase		Length of stay at hospital			P¹
		Ambulation <16 h	Ambulation ≥16 h	Total (n = 381)	
I	mean ± SD	63.3 ± 33.2 (n = 49)	90.4 ± 76.8 (n = 63)	78.5 ± 62.9 (n = 112)	0.008
	median [IQR]	51.4 [48.1–74.7] (n = 49)	58.3 [51.5–98.9] (n = 63)	53.5 [50.5–75] (n = 112)	
II	mean ± SD	58.8 ± 34.4 (n = 87)	85.2 ± 46.5 (n = 59)	69.5 ± 41.7 (n = 146)	<0.001
	median [IQR]	50.7 [46.3–55.9] (n = 87)	72 [51–100.1] (n = 59)	52.2 [48.8–73.9] (n = 146)	
III	mean ± SD	55.9 ± 29.6 (n = 77)	69.4 ± 36 (n = 44)	60.8 ± 32.6 (n = 121)	<0.001
	median [IQR]	50.1 [48.2–53] (n = 77)	54.7 [51.3–75.5] (n = 44)	51.6 [49–69.1] (n = 121)	
Total	mean ± SD	58.8 ± 32.4 (n = 213)	83 ± 58.2 (n = 166)	69.4 ± 47 (n = 379)	<0.001
	median [IQR]	50.8 [48–55.5] (n = 213)	68.1 [51.1–95.5] (n = 166)	52.4 [49.2–73.7] (n = 379)	

(1)Student’s *t*-test.

On global analysis, we drew a parallel between the patients who had begun ambulation before or after 16 h since surgery. The mean length of hospital stay was 83 h for the participants who began ambulation after 16 h since surgery and 58.8 h for the ones who began ambulation before 16 h had elapsed.

In phase II, the mean length of hospital stay was 85.2 h for the participants who began ambulation after 16 h since surgery versus 58.8 h for those who began ambulation before 16 h had elapsed.

In phase III, the difference remained significant; the mean length of hospital stay was 69.4 h for the participants who began ambulation after 16 h since surgery versus 55.9 h for the ones who began ambulation before 16 h had elapsed (p < 0.001).

The length of hospital stay was shorter for participants who began ambulation before 16 h since surgery in phases I (63.3 h), II (58.8 h) and III (55.9 h).

These data indicate that early ambulation starting before 16 h since surgery had elapsed might reduce the length of hospital stay, thus optimising discharge.

### 3.2 Duration of initial ambulation

We analysed the duration of initial ambulation in minutes relative to all the other included variables.

There was no difference in the duration of initial ambulation as a function of access route or magnitude of surgery. In phase I, there was no difference in the duration of initial ambulation as a function of the access route (abdominal: 7.4 min; hysteroscopic: 6.9 min; laparoscopic: 9.2 min; breast: 8.8; vaginal: 12 min; p = 0.963).

The access route did not influence the duration of initial ambulation in phases II (p = 0.246) or III (p = 0.814). The same was the case for magnitude of surgery in phases II (p = 0.735) and III (p = 0.282). In other words, a longer duration of ambulation was not influenced by the access route or magnitude of surgery in any of the study phases.

### 3.3 Walked distance during the IPO and on PO1

In addition to the time to onset of ambulation and duration of ambulation, we also analysed the walked distance. The distance walked on PO1 increased over the study phases relative to phase I.

In phase I, the participants walked a mean of 77.4 m on PO1. Following the implementation of the protocol, the distance walked increased to 292.6 m in phase II, followed by a slight decrease in phase III that was still longer than the distance walked in phase I (mean of 233 m; p < 0.001).

Participants who underwent intermediate surgery walked 181 m on PO1 in phase I, 309.7 m in phase II and 246.8 m in phase III. The distance walked was considerably longer in phases II and III compared with phase I (p < 0.001).

In phase II, the physical therapy protocol that encouraged all patients to walk—i.e., to perform early ambulation regardless of the access route and magnitude of surgery—had already been implemented. Comparison of phase II to phase I revealed a considerable increase in the walked distance among patients subjected to all types of surgeries and corresponding access routes, i.e., abdominal, hysteroscopic, laparoscopic, breast and vaginal (p < 0.001).

As shown in Table 4, regarding the participants who underwent procedures with abdominal access routes, the distance walked on PO1 increased from 58.4 m in phase I to 168 m in phase II (p = 0.030). In regard to the participants subjected to procedures involving hysteroscopy or an endovascular access route, the walked distance increased from 53.2 m in phase I to 303.4 m in phase II (p < 0.001). For those who underwent procedures with breast access, the walked distance increased from 65 m in phase I to 309.6 m in phase II (p < 0.001).

**Table 4:** Correlation of walked distance with access route and magnitude of surgery.

Variables	PHASE I	PHASE II		PHASE III		p value			
	Amb PO1 metres	Amb PO1 metres	Amb IPO metres	Amb PO1 metres	Amb IPO metres	PO1			IPO
	N = 85	N = 178	N = 155	N = 130	N = 139	I vs. II	I vs. III	II vs. III	II vs. III
**Access route**									
Abdominal	58.4 ± 46.1	168 ± 22	67 ± 52	193.1 ± 84.1	51.9 ± 45.1	0.030	<0.001	0.780	0.747
	n = 16	n = 16	n = 16	n = 15	n = 15				
Hysteroscopic or endovascular	53.2 ± 61.8	303.4 ± 174.8	89.1 ± 131.7	251.8 ± 125.9	70.9 ± 51.9	<0.001	<0.001	0.158	0.455
	n = 14	n = 61	n = 43	n = 29	n = 28				
Laparoscopic	118 ± 120.3	249 ± 148.2	49.3 ± 82.1	232 ± 93	84.4 ± 78.4	0.001	0.001	0.660	0.152
	n = 19	n = 24	n = 22	n = 20	n = 19				
Breast	65 ± 59.3	309.6 ± 156.6	94.9 ± 129	258.6 ± 116.5	55.3 ± 77.6	<0.001	<0.001	0.061	0.058
	n = 22	n = 59	n = 56	n = 48	n = 58				
Vaginal	89.5 ± 70.5	260.2 ± 106	92.8 ± 117.5	168.7 ± 85.6	71.8 ± 80.8	<0.001	0.006	0.007	0.532
	n = 14	n = 18	n = 18	n = 19	n = 19				
**Magnitude of surgery**									
Major	111.1 ± 94.2	235 ± 83.1	41.1 ± 68.6	186.3 ± 98.3	89 ± 93.9	<0.001	<0.001	0.048	0.018
	n = 16	n = 45	n = 38	n = 21	n = 29				
Intermediate	181.2 ± 131.8	309.7 ± 167.7	99.9 ± 131.7	246.8 ± 111.8	58.6 ± 61.3	<0.001	<0.001	0.001	0.003
	n = 60	n = 121	n = 106	n = 102	n = 101				
Minor	116.5 ± 90.2	348.6 ± 225.7	103.2 ± 150.2	158.7 ± 101.2	60 .4 ± 57.4	0.002	0.116	0.05	0.439
	n = 9	n = 12	n = 11	n = 7	n = 9				

Amb PO1: ambulation on postoperative day 1; Amb IPO: ambulation during the immediate postoperative period.

### 3.4 Pain, nausea and vomiting and complications

We compared the occurrence of pain, nausea and vomiting between study phases. Pain and nausea significantly improved along the study phases (p < 0.001). The number of pain episodes on PO1 decreased from 0.57 in phase I to 0.34 in phase II and 0.26 in phase III (p < 0.001). The rates of postoperative complications were only 2% in phase I, 3.2% in phase II and 0.53% in phase III (p = 0.24).

## 4 Discussion

The availability of perioperative care protocols can improve recovery after general surgery, and there is strong evidence for benefits following gynaecological surgery. Optimisation of postoperative recovery is influenced by many different factors, such as occurrence of pain, nausea and vomiting, paralytic ileus, tiredness and sleep disorders. The principles of fast track surgery include several aspects, such as providing patients careful preoperative education and information on pre-, peri- and postoperative care, safe and short-acting anaesthesia and pain relief, nutritional care and early mobilisation [20,21].

We are aware that early mobilisation is only part of this hospital discharge optimization project. In our research, in addition to early mobilization, we introduced an abbreviation for fasting, early feedback, antibiotic-prophylaxis, abolition of colonic preparation in elective colorectal surgeries, reduction of perioperative use of intravenous fluids, and restricted use of nasogastric tube and abdominal drains. This study specifically analyzed the impact of physical therapy care, and was responsible for a great change in the care of patients hospitalized at our Hospital. We believe it is very important to highlight and publicize these results, demonstrating that simple physiotherapeutic care, such as early ambulation, can have a significant impact on the surgical outcome, with a low implementation cost.

The ORIGAMI Project developed a protocol to optimise admissions to the gynaecology ward of HSP. The physical therapy staff implemented an instruction and intervention protocol to be applied throughout the patients’ stay at the unit (pre- and immediate postoperative periods) in addition to answering questions from patients regarding their recovery and early mobilisation.

The physical therapy protocol included instruction to be given before surgery and emphasised the importance of early mobilisation and prophylaxis during the immediate postoperative period. Such instruction is believed to dispel doubts regarding the procedures that will be performed, such as transfer to bed and mobilisation [[22], [23], [24]].

The time to onset of ambulation decreased in phase II, from 16 to 13 h, while 25% of the participants began ambulation approximately 6 h following surgery. Ambulation 6 h after gynaecological surgery characterises ultra-early ambulation and was the result of protocol optimisation and the fact that the entire staff was available. Certainly, the participants benefited from the optimisation of early ambulation.

In phase I, the mean time to onset of ambulation was 16 h. This result was used as a reference for all analyses of whether early ambulation contributes to a shorter length of hospital stay. Thus, we sought to establish whether onset of ambulation before or after 16 h since surgery had any impact. The mean length of hospital stay was 83 h for the participants who began ambulation after 16 h since surgery versus 58.8 h for patients who began ambulation before 16 h had elapsed. We emphasise that onset of ambulation 3 h earlier was associated with a shorter hospital stay, with a difference of approximately 24 h.

Analysis of the distances walked during the IPO and on PO1 showed that the data gathered confirms the efficiency of the physical therapy protocol based on the following:

• The distance walked increased following the implementation of the protocol
• Independent of the access route and magnitude of surgery, all the participants in phases II and III—i.e., after the protocol was implemented—were encouraged to walk longer distances
• Early ambulation led to longer walked distances
• Longer walked distances led to shorter hospital stays
• Occurrence of pain resulted in shorter walked distances and indicated that hospital stay might be longer
• The longer the surgery elapsed time, the shorter the distance walked

Regardless of the access route and magnitude of surgery, all participants in phases II and III were encouraged to walk earlier and longer distances.

The early ambulation protocol recommends that patients should start ambulation before 16 h has elapsed since surgery. The results of the present study showed that the participants who began ambulation early walked longer distances. In addition to the fact that patients who began ambulation early walked longer distances, their hospital stays were also shorter.

It is believed that optimisation of bed-leaving improves recovery and minimises the effects of immobilisation syndrome. As a result, patients recover their normal functioning faster and with systemic benefits. While walking longer distances was positively associated with a shorter hospital stay, the duration of ambulation was not a significant factor. This led us to infer that it is better for patients to begin ambulation early and walk longer distances regardless of the duration of ambulation.

Early ambulation improves blood circulation, reduces the occurrence of infection and uterine bleeding, improves respiratory and circulatory function, prevents complications and relieves pain [25]. As a care delivery optimisation strategy, in the past century, patients were instructed not to remain in bed for more than 48 h [[26], [27], [28], [29], [30], [31]].

The ORIGAMI protocol encourages starting ambulation as soon as the clinical conditions of the patient allow for this practice, approximately 6 h following surgery.

## 5 Conclusion

A physical therapy intervention protocol targeting patients admitted to the gynaecological ward of HSP for gynaecological surgery was implemented.

Early ambulation shortened the length of hospital stay, and optimisation of early ambulation reduced the occurrence of episodes of pain, nausea and vomiting.

The distance walked significantly increased from one study phase to the next. This increase in the distance walked was due to the optimisation of the physical therapy protocol, as all participants were encouraged to walk regardless of the magnitude of surgery and the access route.

## Conflicts of interest

There are no financial and personal relationships with other persons or organizations that may inappropriately influence (skew) our work.

There are no potential conflicts of interest.

## Sources of funding

This study has no funding source, there were no sponsors in this study.

## Ethical approval

The present study was submitted to the evaluation and approved by the Research Ethics Committees (CEP), under number 751406, consubstantiated opinion 1,763,134, XXXX and Hospital XXXXX, with Single Research Registry Identifying: Registration search numbers4276.

The present investigation does not present additional risk or discomfort for included patients. All the procedures and interventions implemented will not happen due to the study itself, but rather as the process of improving the quality of Hospital care. All measures are proven beneficial in the light of current knowledge.

The ethical principles in the human sciences are related to the respect to the autonomy of the research subjects, their privacy and protection of data, as well as to avoid damages.

## Consent

Data on patients and groups were submitted to an ethics report and full consent and are documented in this paper.

Written consent is available for review by the editor-in-chief of this journal upon request.

This research was approved by the ethics and research committee of this institution and it was presented justification for not obtaining the informed consent term for each patient. For, it is understood that this work is observational and from this research we will implement improvements in the gynaecology ward.

Patients have the right to privacy. Patient and volunteer names, first names or serial numbers will not be used. Images of patients or volunteers will not be used to inform themselves that the information is scientific and explicit has been given as part of the consent. If the consent is subject to publication, the Chief Editor must be sure of all health conditions.

When consent is given, identification details are omitted if they are not essential.

All patients hospitalized for gynaecological surgeries sign the term HOSPITAL CONSENT, which allows the use of information from the hospital to improve the hospital service.

## Author contribution

Ébe dos Santos Monteiro Carbone, Maria Gabriela Baumgarten Kuster Uyeda, Mayara Ronzini Takaki, Marair Gracio Ferreira Sartori: Conceptualization

Ébe dos Santos Monteiro Carbone, Marair Gracio Ferreira Sartori: Methodology

Ébe dos Santos Monteiro Carbone, Maria Gabriela Baumgarten Kuster Uyeda, Mayara Ronzini Takaki, Marair Gracio Ferreira Sartori: Validation

Ébe dos Santos Monteiro Carbone, Marair Gracio Ferreira Sartori: Formal Analysis

Ébe dos Santos Monteiro Carbone: Investigation

Ébe dos Santos Monteiro Carbone, Marair Gracio Ferreira Sartori: Resources

Ébe dos Santos Monteiro Carbone, Maria Gabriela Baumgarten Kuster: Data Curation

Ébe dos Santos Monteiro Carbone, Marair Gracio Ferreira Sartori: Writing – Original Draft

Ébe dos Santos Monteiro Carbone, Marair Gracio Ferreira Sartori: Writing – Review & Editing

Ébe dos Santos Monteiro Carbone, Maria Gabriela Baumgarten Kuster Uyeda, Mayara Ronzini Takaki, Marair Gracio Ferreira Sartori: Visualization

Ébe dos Santos Monteiro Carbone, Marair Gracio Ferreira Sartori: Supervision

Ébe dos Santos Monteiro Carbone, Marair Gracio Ferreira Sartori: Project Administration.

Marair Gracio Ferreira Sartori: Funding Acquisition.

## Registration of research studies

Research Registry Identifying: Registration search numbers4276.

## Guarantor

This survey had no guarantor or people who contributed financially.

All authors accept responsibility for the work or conduct of the study and have access to the data up to the time of publication.

## Provenance and peer review

Not commissioned, externally peer reviewed.
